# Supplementary material for: Identification and functional analysis of non-coding regulatory small RNA FenSr3 in Bacillus amyloliquefaciens LPB-18
Source: PeerJ. 2023 May 15;11:e15236. doi: 10.7717/peerj.15236 (PMC10194069; doi:10.7717/peerj.15236)
Supplement: Supplemental Information 4 [file peerj-11-15236-s004.zip › KO/CK-vs-T1_map/map04217.html]

KEGG PATHWAY: Necroptosis - Reference pathway


|  |  |
| --- | --- |
| **Necroptosis - Reference pathway** |  |

[
Pathway menu
| Organism menu
| Pathway entry
| Show description
| User data mapping
]

Necroptosis is a programmed form of necrosis. It can be initiated by different stimuli, such as tumor necrosis factor (TNF), TNF-related apoptosis-inducing ligand (TRAIL), Fas ligand (FasL), interferon (IFN), LPS, viral DNA or RNA, DNA-damage agent and requires the kinase activity of receptor-interacting protein 1 (RIPK1) and RIPK3. Its execution involves ROS generation, calcium overload, the opening of the mitochondrial permeability transition pore, mitochondrial fission, inflammatory response and chromatinolysis. Necroptosis participates in many pathogenesis of diseases, including neurological diseases, retinal disorders, acute kidney injury, inflammatory diseases and microbial infections.

|  |  |  |
| --- | --- | --- |
| Reference pathway | 184% 150% 122% 100% 82% 67% 55% | 图片下载 |
